# Gene Expression Dynamics of Sugar Metabolism and Accumulation During Fruit Ripening in *Camellia drupifera*

**DOI:** 10.3390/plants14050817

**Published:** 2025-03-05

**Authors:** Xue Sun, Muhammad Zeeshan Ul Haq, Ya Liu, Dongmei Yang, Huageng Yang, Yougen Wu

**Affiliations:** School of Breeding and Multiplication (Sanya Institute of Breeding and Multiplication), School of Tropical Agriculture and Forestry, Hainan University, Sanya 572025, China

**Keywords:** *Camellia drupifera*, fruit development, sugar metabolism, RNA-seq

## Abstract

*Camellia drupifera*, a valuable woody oil crop, holds significant ecological, economic, and medicinal importance. Its seed maturation involves intricate physiological changes, particularly the interplay between oil biosynthesis and sugar metabolism. This study investigates sugar accumulation and the expression dynamics of sugar metabolism-related unigenes during three key developmental stages of *C. drupifera* fruit: the nutrient synthesis stage (NS), fat accumulation stage (FA), and maturation stage (MS). The findings reveal distinct differences in sugar content and regulatory mechanisms across the stages. The NS stage emerges as a critical period for sugar metabolism, characterized by peak levels of soluble sugars and fructose alongside a significantly elevated expression of sugar metabolism-related unigenes. The significant correlation between sucrose content and gene expression suggests a crucial role of carbohydrates in fruit maturation. Transcriptomic analysis identified key differentially expressed unigenes (DEGs) in sugar metabolism pathways, which qRT-PCR further validated. These results offer novel insights into the molecular mechanisms regulating sugar metabolism during *C. drupifera* fruit development. At the same time, it provides a theoretical basis for the genetic improvement and effective utilization of other oil crops, supporting their broader agricultural and industrial applications.

## 1. Introduction

*Camellia drupifera (C. drupifera*), commonly known as oil tea, is an evergreen shrub from the *Theaceae* family, native to China [1]. As a significant woody oilseed crop, it produces edible *Camellia* oil from its seeds [2]. China accounts for approximately 95% of the global *Camellia* oil production [3]. *Camellia* oil is highly valued for its medicinal and culinary benefits. It is characterized by a high content of unsaturated fatty acids, such as oleic acid, linoleic acid, and palmitic acid while exhibiting a low concentration of saturated fatty acids [4,5]. Its fatty acid profile resembles olive oil, earning it the nickname “Oriental olive oil” [6,7]. The fruit of *C. drupifera* primarily contains soluble sugars such as sucrose, fructose, and glucose, with sucrose being the primary storage carbohydrate [8]. *C. drupifera*, a distinct ecological variety cultivated in tropical regions, thrives on Hainan Island. Due to the island’s unique geographical and climatic conditions, this variety is recognized as a distinct genetic resource superior to other *C. oleifera* varieties found on the Chinese mainland [9]. Beyond its economic significance, the cultivation of *C. drupifera* plays a crucial role in soil conservation and biodiversity preservation, contributing to ecological stability and sustainable development [10,11].

Photosynthesis is a biochemical process through which green plants, algae, and certain bacteria capture solar energy to convert inorganic substrates, primarily carbon dioxide (CO_2_) and water (H_2_O), into organic compounds, predominantly carbohydrates. During this process, oxygen (O_2_) is released as a byproduct. [12]. The synthesis, transport, storage, and utilization of carbohydrates form a comprehensive network that supports the entire growth cycle of plants [13]. During photosynthesis, sugars are synthesized in the leaves and transported to other plant parts, including the fruits [14]. Sucrose, the primary product of photosynthesis, is the foundation for C synthesis and is integral to storing proteins, starch, and fats, which are essential for plant growth [15]. Sugars are fundamental for energy provision and growth promotion in higher plants and function as signaling molecules that regulate metabolism and defense responses [16]. Sugar metabolism plays a central role in carbohydrate metabolism, with its intricate regulatory network integrating internal and external factors to maintain plant growth and development [17]. In summary, photosynthesis is critical for converting light energy into chemical energy stored in carbohydrates, essential for plant growth and development [18]. The dynamic processes of sugar synthesis, transport, and utilization underscore the importance of carbohydrates in maintaining physiological balance within plants [19].

In sucrose biosynthesis, fructose-6-phosphate (F-6-P) and uridine diphosphate glucose (UDP-G) serve as substrates for sucrose phosphate synthase (*SPS*), which catalyzes the formation of sucrose-6-phosphate (S-6-P). This intermediate is subsequently hydrolyzed by sucrose phosphate phosphatase (*SPP*) to produce sucrose [19]. Sucrose synthase (*SUS*) also catalyzes a reversible reaction, cleaving sucrose into fructose and UDP-G, thereby contributing to both sucrose synthesis and degradation [20,21,22,23]. This highlights the complexity of sucrose metabolism and the crucial roles of various enzymes in carbohydrate synthesis and degradation. Research has shown that gene expression was related to sucrose synthesis peaks during the early stages of fruit development, coinciding with high sucrose accumulation. As the fruit matures, there is a significant increase in the expression of unigenes involved in starch degradation and hexose transport, reflecting changes in sugar metabolism as the fruit develops [24]. A study by Yu et al. (2021) demonstrated that genes associated with sucrose synthesis and degradation are differentially expressed at various stages of fruit development, with key enzymes such as *SPS*, invertase, and different transport proteins being identified as critical regulators of sugar accumulation [25]. Wang et al. (2020) observed that early development in *Citrullus lanatusis* is marked by high sucrose synthesis, while later stages involve increased expression of genes related to sugar degradation and transport, particularly those encoding *SPS*, invertase, and sugar transport proteins [26]. The decline in sucrose content during *C. lanatusis* maturation is likely a response to the energy demands and metabolic regulation required for fruit growth and maturation. Wyatt et al. (2016) sequenced the transcriptomes of “Sweet REBA” and “Lady Godiva” gourds, identifying trends in gene expression associated with sucrose and starch metabolism throughout fruit development [27]. Additionally, Apriyanto et al. (2022) reported that high-yield and medium-yield oil palm varieties exhibited significantly higher sucrose levels than low-yield varieties [28]. This was correlated with starch characteristics, suggesting that sucrose and starch metabolism are pivotal in the biosynthesis of oil palm fruits [28].

While numerous sugar transport and metabolism unigenes have been identified in other species, the molecular mechanisms regulating sugar metabolism in *C. drupifera* fruit remain poorly understood. Sugar is an essential precursor for lipid synthesis. Therefore, identifying key unigenes involved in sugar transport and metabolism during the developmental stages of *C. drupifera* fruits is critically important for understanding lipid biosynthesis in later stages of development. While current research has primarily focused on the molecular mechanisms underlying lipid synthesis in *C. drupifera*, studies examining the influence of carbohydrate metabolism on lipid production remain limited. This study aims to (1) systematically analyze the dynamic changes in sugar content and (2) elucidate the associations and differences in sugar metabolism pathways across various maturation stages of *C. drupifera* fruits, focusing on identifying key regulatory unigenes. The findings comprehensively explain the mechanisms governing sugar metabolism in *C. drupifera*. Moreover, the results serve as a foundation for targeted genetic engineering strategies, contributing to the improved development and utilization of resources within this valuable genus.

## 2. Results

### 2.1. Developmental Changes in Phenotypic and Economic Traits

**Figure 1:**
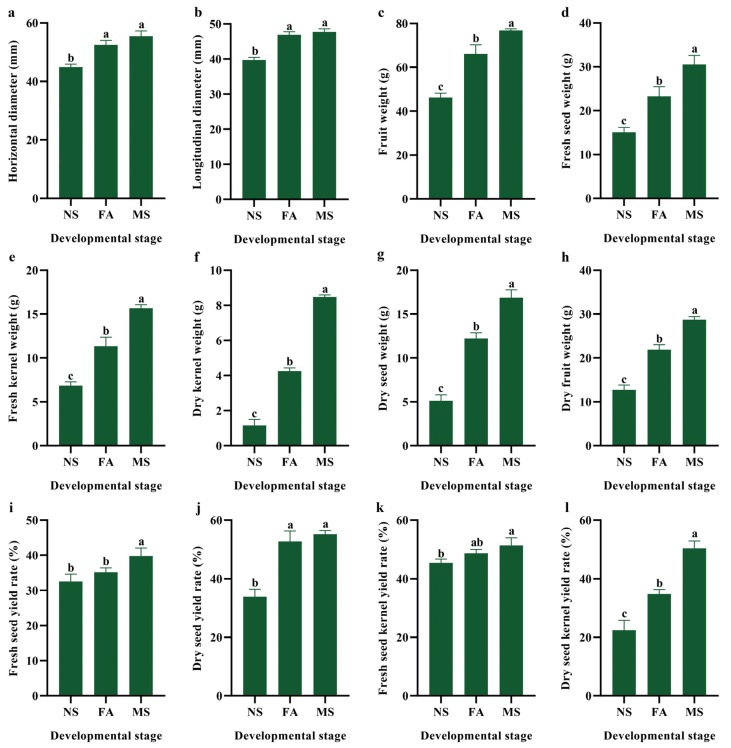
The changes in phenotypic characteristics and economic traits during the three developmental stages of *C. drupifera*. The data are expressed as the mean ± S.D. Small alphabets indicated significant variations in three developmental stages (*p* < 0.05). (**a**) Horizontal diameter (mm); (**b**) Longitudinal diameter (mm); (**c**) Fruit weight (g); (**d**) Fresh seed weight (g); (**e**) Fresh kernel weight (g); (**f**) Dry kernel weight (g); (**g**) Dry seed weight (g); (**h**) Dry fruit weight (g); (**i**) Fresh seed yield rate (%); (**j**) Dry seed yield rate (%); (**k**) Fresh seed kernel yield rate (%); (**l**) Dry seed kernel yield rate (%).

The results indicated a clear progression in the physical and nutritional characteristics of *C. drupifera* as it develops from the nutrient synthesis (NS), fat accumulation (FA), and maturation (MS) stages (Figure 1 and Table S1). The horizontal diameter of the fruit increased by 16.9% from NS to FA and 23.5% from NS to MS (Figure 1a), while the longitudinal diameter showed a similar trend with increases of 18.0% and 20.1%, respectively (Figure 1b). Regarding fruit weight, a notable increase of 43.0% was observed from NS to FA, which further increased to 66.1% at the MS stage, indicating substantial fruit mass gain as the fruit matures (Figure 1c). The fresh seed weight increased by 54.4% at FA and an impressive 102.7% at MS, relative to NS, showing that seed development progresses through these stages (Figure 1d). Fresh kernel weight followed a similar pattern, with a 65.6% increase from NS to FA and a significant 129.2% increase from NS to MS (Figure 1e). The most significant increase was observed in the dry kernel weight, which increased by 265.6% from NS to FA and a remarkable 628.7% from NS to MS, highlighting the substantial dry matter accumulation in the kernel as the fruit matures (Figure 1f). These findings highlighted the significant growth and nutrient accumulation of *C. drupifera*, particularly in the kernel, throughout its developmental stages, with the MS exhibiting the highest values across all measured parameters. Dry seed weight increased significantly, with FA reaching 238.6% and MS reaching 328.9% of the initial value at NS (Figure 1g and Table S2). Similarly, dry fruit weight increased by 72.2% at FA and 126.8% at MS compared with NS (Figure 1h). The fresh seed yield rate showed a reasonable increase, with FA and MS reaching 107.9% and 121.9% of NS, respectively (Figure 1i). The dry seed yield rate displayed a notable increase, with FA being at 155.4% and MS at 162.8% of the NS value (Figure 1j). A fresh seed kernel yield rate also increased moderately, reaching 107.3% at FA and 113.2% at MS (Figure 1k). The most significant increase was observed in the dry seed kernel yield rate, which increased to 155.3% at FA and 224.7% at MS compared with NS (Figure 1l). These findings indicated that as the fruit matures, there is a significant accumulation of biomass, particularly in the dry seed and kernel, reflecting the plant’s enhanced nutrient and resource allocation towards the end of its development. The MS exhibited the highest values across most parameters, underscoring this stage as the peak of nutrient synthesis and accumulation in *C. drupifera*.

### 2.2. Physicochemical Analysis Changes

**Figure 2:**
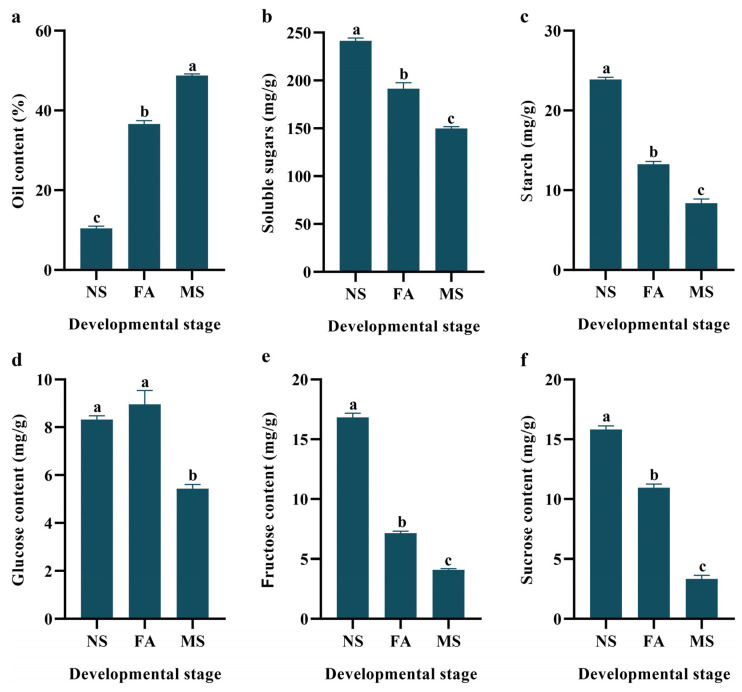
Changes in physiological indicators during the three developmental stages of *C. drupifera*. The data are expressed as the mean ± S.D. Small alphabetical letters indicated significant variations in three developmental stages (*p* < 0.05). (**a**) Oil content (%); (**b**) Soluble sugars (mg/g); (**c**) Starch (mg/g); (**d**) Glucose content (mg/g); (**e**) Fructose content (mg/g); (**f**) Sucrose content (mg/g).

The analysis of *C. drupifera* at different developmental stages, NS, FA, and MS, revealed significant physiochemical analysis changes (Figure 2 and Table S3). Oil content increased significantly, reaching 351.1% of the NS level at FA and peaking at 467.5% at MS, indicating substantial oil accumulation as the fruit matures (Figure 2a). In contrast, soluble sugars and starch levels declined as the fruit developed, with soluble sugars decreasing to 20.7% of NS at FA and 38.0% at MS (Figure 2b), while starch decreased to 44.5% at FA and further to 65.0% at MS (Figure 2c), suggesting that these carbohydrates are used as energy sources for oil and protein synthesis. Glucose levels increased by 7.7% during FA, reaching 107.7% of the NS level, followed by a decline to 65.4% in MS (Figure 2d). Fructose content consistently decreased, dropping to 42.4% during FA and further declining to 24.3% in MS relative to NS (Figure 2e). Conversely, the sucrose level in FA increased to 227.3% compared with the NS stage and a further 372.7% during MS (Figure 2f). These results highlighted the dynamic regulation of sugar metabolism during fruit development, with distinct variations across the three developmental stages.

### 2.3. Correlation Analysis

**Figure 3:**
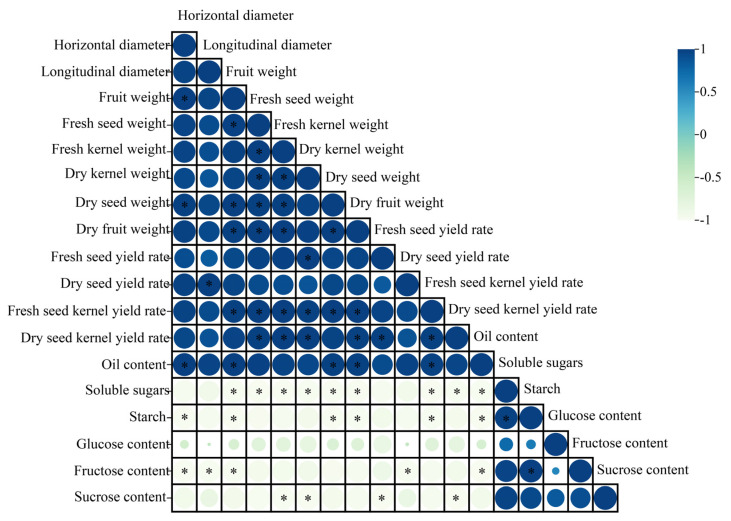
Correlation analysis between economic traits and physicochemical indicators, with a significance level set at * *p* < 0.05.

A Pearson correlation analysis was performed using IBM SPSS Statistics 25 software to examine the relationships between economic traits and physicochemical indicators, with a significance level set at *p* < 0.05 (Figure 3, Table S4). The results revealed a positive correlation between oil content and all economic traits, while a negative correlation was observed with physicochemical indicators. Specifically, horizontal diameter, fruit weight, dry seed weight, dry fruit weight, and fresh seed yield rate were significantly positively correlated with oil content. In contrast, soluble sugars, starch, and fructose showed significant negative correlations with oil content. Furthermore, soluble sugars were positively correlated with starch, and starch was positively correlated with fructose. The correlations among fructose, glucose, and sucrose were positive but not statistically significant.

### 2.4. Transcriptome and Differential Unigene Analysis

**Figure 4:**
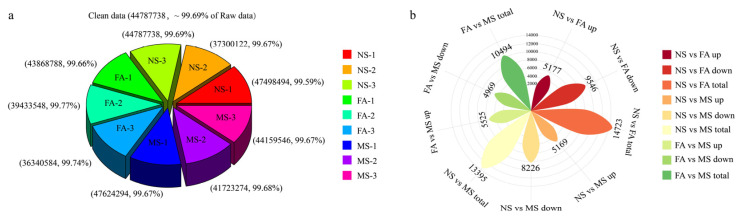
RNA sequencing data of *C. drupifera* at different developmental stages. Clean data generated by sequencing (**a**). The number of DEGs (**b**). Abbreviations: NS, nutrient synthesis stage; FA, fat accumulation stage; MS, maturity stage.

**Figure 5:**
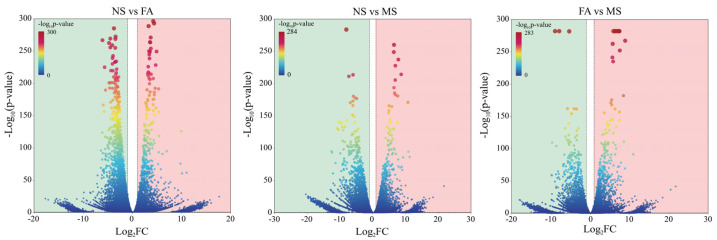
Gradient volcano diagram of the number of *C. drupifera* DEGs at different developmental stages.

**Figure 6:**
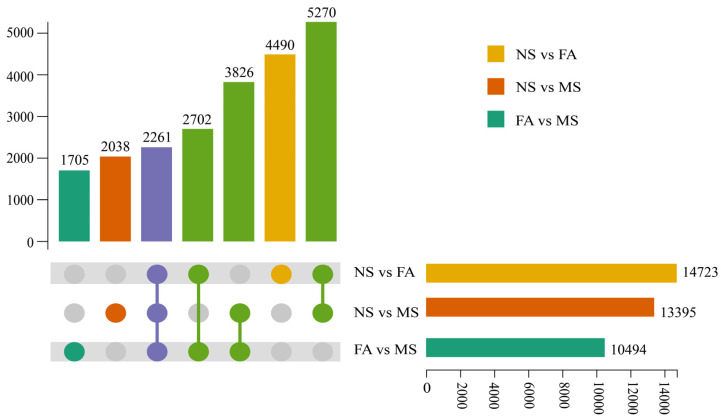
Upset plot of the DEGs in *C. drupifera* at different developmental stages.

To uncover the molecular networks regulating the development of *C. drupifera* fruit, nine cDNA libraries were constructed using samples from the NS, FA, and MS stages. The clean reads from each library accounted for more than 99% of the total raw reads, ensuring high-quality data (Figure 4a). The reads were assembled and clustered into transcripts based on sequence similarity using Trinity assembly technology, resulting in 85,288 unigenes. The lengths range from 201 bp to 15,601 bp, with an N50 value of 15,086 bp and an average length of 1401 bp. All assembled unigenes were aligned against four major databases (NR, SwissProt, KOG, and KEGG) to obtain detailed descriptive information about the genes and further analyze their involved biological functions. The results showed that a total of 85,288 unigenes were successfully annotated, of which 44,402 unigenes were annotated in the NR database and 40,291 unigenes were annotated in the KEGG database. A homology analysis revealed that 88.87% of the unigenes were successfully mapped to the *C. drupifera* genome through alignment with the Nr database. A principal component analysis (PCA) was performed based on the gene expression profiles. However, the MS-1 sample was excluded from further analysis due to its low mapping rate. Pairwise comparisons of samples across the three developmental stages of NS vs. FA, NS vs. MS, and FA vs. MS identified 14723, 13395, and 10494 differentially expressed unigenes (DEGs), respectively (Figure 4b). The DEG patterns in *C. drupifera* seeds were visualized using a volcano plot (Figure 5 and Table S5). Specifically, 5177 upregulated and 9546 downregulated unigenes were identified in NS vs. FA, 5169 upregulated and 8226 downregulated unigenes in NS vs. MS, and 5525 upregulated and 4969 downregulated unigenes in FA vs. MS. Furthermore, 4490, 2038, and 1705 unique unigenes were identified in the NS vs. FA, NS vs. MS, and FA vs. MS comparisons, respectively (Figure 6).

### 2.5. Functional Annotation of DEGs

**Figure 7:**
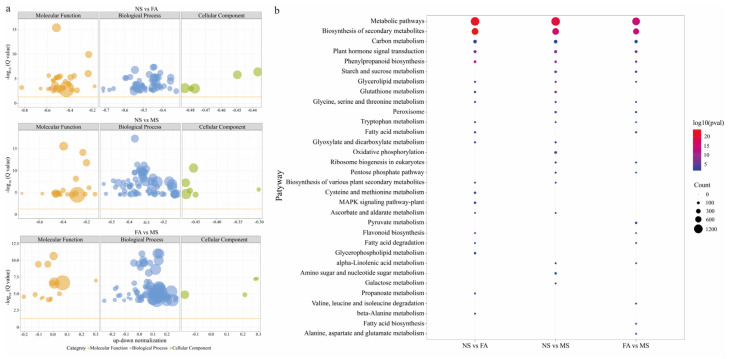
Enrichment analysis of DEGs. GO classification and distribution (**a**). Enriching the most significant 20 KEGG pathways of NS vs. FA, NS vs. MS, and FA vs. MS (**b**).

The Gene Ontology (GO) classification offered insights into the properties of gene products across three categories: biological processes, cellular components, and molecular functions. In this study, 11,794 unigenes were categorized into 54 functional subclasses (Figure 7a). Most annotations pertain to biological processes, with a strong focus on cellular processes. Cell-related annotations are the most prominent at the cellular component level. Binding and catalytic activity are the predominant categories for molecular functions. These findings suggested the identified unigenes primarily involved fundamental biological regulation and metabolic activities shared across plants. The pathway-based analysis offers a deeper understanding of gene functions and interactions. The 3416 unigenes were mapped to 140 biological pathways using the KEGG database. The KEGG enrichment analysis of these DEGs provides insights into the molecular mechanisms underlying key metabolic processes in *C. drupifera* fruit development. The DEGs were predominantly enriched in metabolic processes, secondary metabolite biosynthesis pathways, carbon metabolism, plant hormone signal transduction, starch and sucrose metabolism, and phenylpropanoid biosynthesis (Figure 7b). The KEGG analysis of DEGs in *C. drupifera* seeds across developmental stages revealed 402, 412, and 316 DEGs associated with carbohydrate metabolism during the NS, FA, and MS stages, respectively (Table S6). These results highlighted the critical roles of these pathways in regulating the physiological and biochemical processes during fruit development in *C. drupifera*.

A pathway enrichment analysis of DEGs (including upregulated and downregulated unigenes) revealed that upregulated unigenes were significantly enriched in biological pathways related to carbohydrate and amino acid metabolism. Conversely, downregulated unigenes were primarily enriched in biological pathways associated with carbohydrate and lipid metabolism. In the context of DEGs related to carbohydrate metabolism, a total of 278 unigenes were identified as upregulated, while 440 unigenes were downregulated (Figure S1). The KEGG enrichment analysis revealed that unigenes associated with pyruvate metabolism, propanoate metabolism, and glyoxylate and dicarboxylate metabolism were significantly upregulated (Table S7). Conversely, downregulated unigenes were predominantly enriched in ascorbate and aldarate metabolism, amino sugar and nucleotide sugar metabolism, pentose phosphate pathway, and starch and sucrose metabolism (Table S8). These findings suggested the activation of energy production and carbon skeleton biosynthesis pathways, concurrent with suppressing pathways involved in antioxidation, saccharide synthesis, and specific carbon metabolic processes within the biological context under investigation. Further statistical analysis of carbohydrate-related DEGs identified the distribution of these unigenes in key sugar metabolism pathways, including glycolysis/gluconeogenesis, starch and sucrose metabolism, and citric acid cycle.

### 2.6. Co-Expression Network Analysis with WGCNA

**Figure 8:**
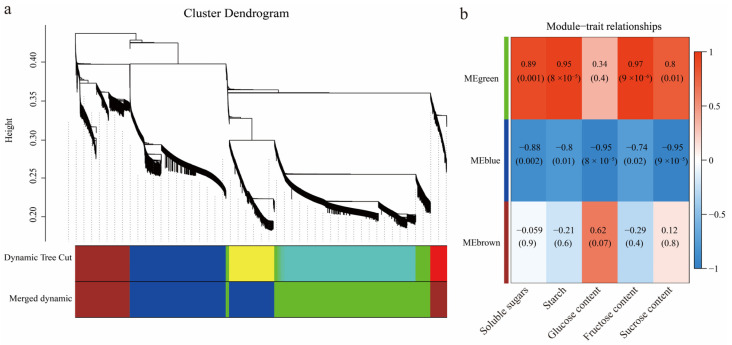
A WGCNA of differential gene expression during *C. druifera* development was performed. Based on consensus topological overlap, hierarchical clustering was used to generate a gene dendrogram, with each branch representing a module and each leaf representing a gene. Each colored row represents a color-coded module containing a set of highly interconnected genes (**a**). Module eigengene relationships with physiological traits and sample relationships are shown (**b**). The numbers in the colored rectangles indicate the number of genes within each module. The color scale on the right displays the range of correlations from negative to positive.

To identify weighted gene co-expression network analysis (WGCNA) modules related to sugar metabolism during *C. druifera* fruit ripening, we constructed a co-expression network utilizing a high-throughput RNA-seq dataset, combined with changes in soluble sugars, starch, glucose, fructose, and sucrose (Figure 8). A total of three distinct modules, encompassing 16,207 non-redundant unigenes, were identified and labeled with different colors, presented in the form of a clustering dendrogram and correlation heatmap. Among these, the MEgreen module (containing 6951 unigenes) exhibited a strong correlation with the accumulation patterns of soluble sugars, starch, glucose, fructose, and sucrose, with absolute correlation coefficients being greater than 0.8 (*p* ≤ 0.01). The highest correlation was observed with fructose (r = 0.97, *p* = 9 × 10^−10^), followed by starch (r = 0.95, *p* = 8 ×10^−5^) and soluble sugars (r = 0.89, *p* = 0.001). Subsequently, a statistical enrichment analysis was performed on the DEGs within the MEgreen module to explore the biological functions of the transcriptome within this module (Table S9). This further identified the distribution of these unique genes in key sugar metabolism pathways, including glycolysis/gluconeogenesis, starch and sucrose metabolism, and the citric acid cycle. Enzymes associated with sugar metabolism, such as *SS*, *SUS7*, *HXK2*, *SPS4*, and *ALDH2B4,* were also identified in previous analyses.

### 2.7. Analysis of Expression Patterns of DEGs

**Figure 9:**
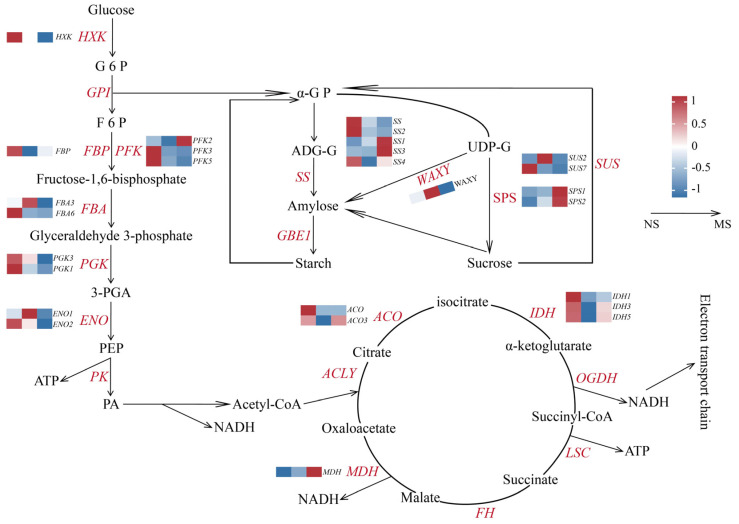
Key unigene expression patterns of carbohydrate metabolism pathways in *C. drupifera* seeds. *FBP*: fructose-1,6-diphosphatase; *PFK*: 6-phosphofructokinase; *FBA*: fructose diphosphate aldolase; *PGK*: phosphoglycerate kinase; *ENO*: enolase; *SS*: sucrose synthase; *WAXY*: grain-bound starch synthase; SPS: sucrose phosphate synthase; *SUS*: sucrose synthase; *IDH*: isocitrate dehydrogenase (NADP+); *MDH*: malate dehydrogenase; *HK*: hexokinase; *GPl*: glucose-6-phosphate isomerase; GBE1: starch-branching enzyme; *PK*: acetoacetate stimulation; *ACLY*: ATP citrate (pre-S) lyase; *OGDH*: 2-oxoglutarate dehydrogenase (E1); *LSC*: succinyl CoA synthase alpha subunit; *FH*: fumarate hydratase.

In this study, we identified and characterized the expression profiles of DEGs associated with key pathways in sugar metabolism (Figure 9 and Table S10), encompassing critical processes such as glycolysis/gluconeogenesis, starch and sucrose metabolism, and the tricarboxylic acid (TCA) cycle. During the three developmental stages of *C. drupifera*, the expression of unigenes encoding key enzymes involved in sucrose and starch metabolism, such as sucrose synthase (*SS*) and sucrose synthase (*SUS*), peaked during the NS stage. Furthermore, the *WAXY* gene, which encodes grain-associated starch synthase, exhibited its highest expression during the FA stage, whereas the sucrose phosphate synthase (*SPS*) gene demonstrated maximal expression in the MS stage. In the glycolysis/gluconeogenesis and TCA cycle pathways, the expression of unigenes encoding key enzymes displayed stage-specific variations. For instance, during the NS stage, unigenes such as fructose-1,6-bisphosphatase (*FBP*), phosphofructokinase (*PFK*), phosphoglycerate kinase (*PGK*), acetyl-CoA carboxylase (*ACO*), and isocitrate dehydrogenase (*IDH*) showed their highest expression levels. In contrast, the expression of the malate dehydrogenase (*MDH*) gene was significantly upregulated during the MS stage. These results highlighted the stage-specific regulation of key metabolic enzymes, suggesting their distinct biological roles in aligning energy metabolism and biosynthetic demands during different growth stages. The results emphasized significant variations in the regulatory mechanisms governing sugar metabolism-related unigenes across developmental stages, reflecting the dynamic energy and substrate requirements of *C. drupifera* throughout its growth and development.

### 2.8. Quantitative Real-Time PCR Analysis of Selected Genes

**Figure 10:**
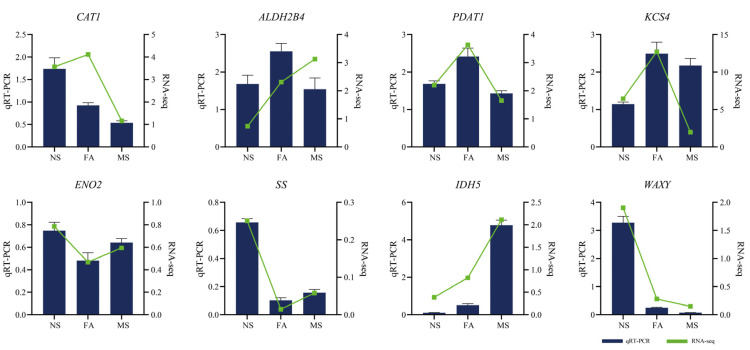
The qRT-PCR was used to validate the DEGs at different developing stages of *C. drupifera* seeds. The figure’s blue sections represent the fold change (FC) values from qRT-PCR results, while the lines represent the FC values from RNA-seq results.

To verify the reliability of transcriptome data, eight DEGs that may be involved in sugar metabolism were randomly selected for quantitative real-time PCR (qRT PCR) analysis (Figure 10). The results demonstrated that the qRT-PCR data were consistent with the RNA-seq findings, thereby confirming the reliability of the transcriptome data generated in this study.

## 3. Discussion

Plant growth, development, and productivity mainly depend on photosynthetic product synthesis, transportation, and distribution [29]. This study measured the fruit traits of *C. drupifera* at different developmental stages and found that as the fruit develops, the fruit size and weight differences gradually increase, especially reaching their highest values at the MS stage. The significant increase in fruit weight indicated physiological growth changes and the accumulation of essential substances necessary for developing *C. drupifera*. The most significant increase was in dry grain weight, which increased by 266.4% from NS to FA and 630.2% from NS to MS. The significant accumulation of this dry matter is crucial for oil yield as it reflects the effective resource allocation of plants during fruit development [30]. Extensive evidence suggests that the correlation between dry matter weight and oil content underscores the importance of optimizing dry matter accumulation to enhance oil production [16,31,32]. Similarly, the transverse and longitudinal diameters of the fruit also exhibit the same trend. These findings aligned with research on Huanglian wood development, highlighting fruit ripening as a complex physiological process influenced by environmental factors such as temperature and water availability [33,34]. The increase in oil content indicated that *C. drupifera* has evolved effective mechanisms for oil synthesis and storage during fruit ripening. Multiple factors may regulate this process, including environmental conditions, genetic characteristics, and hormone signaling pathways [35].

Genes often participate in biological processes through coordinated expression. Therefore, WGCNA was utilized to construct co-expression networks, identifying key unigenes involved in sugar metabolism during *C. drupifera* fruit maturation, including SUS, SPS, and SS. Soluble sugars provide energy and act as signaling molecules that regulate physiological processes, supporting lipid accumulation [8,36]. The soluble sugars in *C. drupifera* fruit were mainly composed of glucose, fructose, and sucrose, and their content and proportion vary among different tree species, different strains of the same tree species, different organs of the same plant, and different stages of enrichment in the same organ [8,37,38,39]. Sugar accumulation is a complex metabolic process regulated by the coordinated activities of sugar synthesis, transport, metabolism, and storage throughout fruit development [40]. The NS and FA stages were critical periods for *C. drupifera* fruit enlargement and oil synthesis and transformation, as the fruit consumes a lot of energy to achieve development and maturity. In this study, the reduction of soluble sugars and starch suggests that they may be mobilized as energy sources for oil synthesis and protein production during seed development. Plant sucrose can act as a metabolite or signaling molecule to initiate signaling pathways, leading to changes in gene expression and physiological adaptation [41]. The synthesis of sucrose requires the synergistic action of unigenes such as sucrose synthase (*SUS*) and sucrose phosphate synthase (*SPS*) [42]. Sucrose levels can also be regulated to increase early activity and promote energy conversion [8]. High concentrations of sucrose provide an important foundation for nutrient accumulation and quality formation during fruit development [37], promoting the transition from sugar metabolism to fat and protein metabolism [43]. By observing the transcription levels and sucrose content of unigenes related to sucrose accumulation, we aim to elucidate the impact of gene expression levels on sucrose accumulation during the development of *C. drupifera* fruits. The *SUS* can catalyze the reverse conversion of sucrose and UDP to UDP-glucose and fructose, playing different roles at different developmental stages [44]. The results showed that the *SUS* gene was highly expressed in the NS or FA stage, but at this time, the sucrose content was lower. This may be due to the fact that at this time, the *SUS* gene mainly acts on the decomposition of sucrose, increasing the activity of its related enzymes, thereby accelerating sucrose decomposition and promoting oil synthesis. Research has shown that the *SUS* gene is believed to be associated with the accumulation and metabolism of sucrose in citrus [45] and apple [46] fruits. Similarly, in *Dendrobium officinale*, with fluctuations in sucrose levels, the *SUS* gene may be negatively correlated with sucrose accumulation [47]. On the contrary, inhibition of the *FaSUS1* gene was found to significantly delay fruit ripening and downregulate sucrose and anthocyanin content in strawberry fruits [48]. In addition, the expression of the *SUS* gene is highly correlated with biomass accumulation. Currently, the function of this gene in *C. drupifera* has not been confirmed, but studies have shown that overexpression of the *SUS* gene can lead to an increase in the weight of *Zea mays* seeds [49], *Oryza sativa* seeds [50], and *Solanum tuberosum* tubers [51]. Compared with NS, the expression of the *SPS* gene in *C. drupifera* fruit is upregulated at the FA and MS stages and reaches its peak at the MS stage. As a key enzyme in sucrose synthesis, *SPS* has a similar effect on sucrose metabolism in fruits such as *Malus × domestica Borkh* [52], *Symplocos paniculate* [53], *Citrus* [19], *Saccharum officinarum* [54], *Prunus persica* [24], etc. At the same time, the synthesis and metabolism of sucrose were closely related to fructose and glucose. The *SPS* not only participates in the synthesis of sucrose but also has the function of decomposing sucrose into fructose and uridine diphosphate glucose to support oil synthesis [36].

Fructose is a monosaccharide that can be easily metabolized into energy or converted into other metabolites to promote growth and accumulate oil. Studies have shown that the decrease in fructose content may be due to the high energy requirements for fruit ripening [55]. In this study, the fructose and glucose content in *C. drupifera* showed the lowest values during the MS stage, indicating that a large amount of reducing sugars were consumed by respiration, and the energy generated was used for various metabolic activities of *C. drupifera*. The increase in glucose content during the FA stage may reflect the enhancement of its photosynthetic activity. The positive correlation between sucrose and oil content indicated that higher sucrose promotes oil accumulation [35], while reducing sugars such as glucose are negatively correlated with oil content [56], which is consistent with the results of this study. This study found that downregulation of the *WAXY* unigene may lead to a significant reduction in starch content in *C*. *drupifera* fruits. This is likely because the *WAXY* gene encodes granule-bound starch synthase (GBSS), a key enzyme in amylose synthesis. The decreased expression level of the *WAXY* unigene directly affects the activity of GBSS, thereby inhibiting amylose synthesis and ultimately reducing the total starch content in the fruits. Meanwhile, studies have shown that reduced starch content may influence lipid synthesis, such as an increase in oil content or changes in fatty acid composition. The unigenes related to sugar metabolism played an important role in regulating the sugar accumulation in *C. drupifera*. There are differences in the effects and mechanisms of various metabolic enzymes on the soluble sugar content and the accumulation of starch, glucose, fructose, and sucrose. Therefore, further analysis can be conducted on the metabolic enzyme action modes and deep molecular mechanisms related to the differences in sugar accumulation among different *C. drupifera* varieties, providing a reference for the influence mechanism of *C. drupifera* fruit traits.

## 4. Materials and Methods

### 4.1. Plant Material

The *C. drupifera* plants were cultivated at the Germplasm Resource Base in Hongmao Town, Qiongzhong City, Hainan Province, China (19°0′56″ N; 109°42′59″ E). The region experiences an annual average temperature of 22–24 °C, an annual average sunshine duration of 1600–2000 h, and an annual rainfall ranging from 2200 mm to 2444 mm. Fresh fruit samples were collected at three developmental stages between August and November 2023: the nutrient synthesis stage (NS; 270 days after pollination), fat accumulation stage (FA; 315 days after pollination), and maturity stage (MS; 360 days after pollination). Nine healthy and vigorous high-yielding individual *C. drupifera* trees were selected for sampling. Every three trees were treated as one biological replicate. In the mid-periphery of the crown of each tree, fruits that were uniform in shape and size, similar in color, and free from mechanical damage and pest or disease infestation were randomly selected for the collection. At each developmental stage, *C. drupifera* fruits were divided into two portions. One portion was air-dried for physicochemical property examination. The other portion was peeled, wrapped in tin foil, immediately snap-frozen in N_2_, and stored at -80 °C for further transcriptome analysis. In the transcriptome analysis, the NS stage was used as the control group to compare the expression changes of sugar metabolism-related unigenes between the FA and MS stages.

### 4.2. Fruit Economic Characteristics Determination

The transverse/horizontal and longitudinal diameters of fresh fruits were measured using a vernier caliper (Biaokang, Shenzhen, China, SL01-3, 0.1 mm), and an electronic balance (Changshu Tianliang Instrument Co., Ltd., Changshu, China LT302B electronic balance, 0.01 g) was used to determine the fresh fruit weight, fresh seed weight, and fresh seed kernel weight. Each fruit part was oven-dried (Shanghai Yiheng Scientific Instrument Co., Ltd., Shanghai, China DHG-9053A), initially deactivated at 105 °C for 15 min, and subsequently baked at 70 °C for 72 h. After drying, the dry seed and kernel weights were measured with an electronic balance.

The following formulas were used to calculate yields:

- Fresh seed yield (%) = (fresh seed weight/single fruit weight) × 100;
- Dry seed yield (%) = (dry seed weight/fresh seed weight) × 100;
- Fresh seed kernel yield (%) = (fresh kernel weight/fresh seed weight) × 100;
- Dry seed kernel yield (%) = (dry kernel weight/dry seed weight) × 100.

Seed kernel oil content was determined using Soxhlet extraction, following the method by Ye et al. [35].

### 4.3. Physicochemical Parameters Measurement

Seed kernel oil content (KOC) was determined using Soxhlet extraction, following the method employed by Ye et al. [35]. The soluble sugar and starch content kit developed by Nanjing Jiancheng Institute of Bioengineering was used to determine the soluble sugar and starch. Using a micro-quantity detection kit developed by Suzhou Greeis Biotechnology Co., Ltd. (Suzhou, China), the contents of sucrose, glucose, and fructose in three samples of *C. drupifera* fruits from Hainan were quantitatively assessed.

### 4.4. RNA Extraction and Transcriptome Library Construction

Total RNA extraction was performed using the TRIzol kit (Invitrogen, Carlsbad, CA, USA), following the manufacturer’s protocol. The quality of the extracted RNA was assessed with an Agilent 2100 Bioanalyzer (Agilent Technologies, Palo Alto, CA, USA) and by ribonuclease-free agarose gel electrophoresis. RNA samples with A260/A280 and A260/A230 ratios greater than 2.0 were deemed suitable for downstream analyses. For mRNA enrichment, oligo (dT) magnetic beads were employed to isolate eukaryotic mRNA, while rRNA depletion using the Ribo-Zero™ magnetic kit (Illumina, San Diego, CA, USA) was used to enrich prokaryotic mRNA. The enriched mRNA was then fragmented using a fragmentation buffer and subsequently reverse-transcribed into cDNA using random primers. Second-strand synthesis was conducted with DNA polymerase I, RNase H, dNTPs, and a reaction buffer. The cDNA fragments underwent purification with a QIAquick PCR extraction kit (Qiagen, Venlo, The Netherlands), followed by end-repair and the addition of an adenine (A) base to their 3′ ends. Illumina sequencing adapters were ligated to the prepared cDNA fragments. After ligation, the products were purified via agarose gel electrophoresis, amplified by PCR, and sequenced using the Illumina NovaSeq 6000 platform Gene Denovo Biotechnology Co., Guangzhou, China.

### 4.5. Bioinformatics Analysis

After sequencing, raw reads were processed using FASTQ (v0.18.0) to remove low-quality reads, resulting in clean reads for downstream analysis. These clean reads were assembled using Trinity (v2.15.1) software. A differential expression analysis between the two groups was conducted using DESeq2, with unigenes meeting the criteria of a false discovery rate (FDR) < 0.05 and an absolute fold change ≥ 2 identified as DEGs. All identified DEGs were annotated by mapping to the Gene Ontology (GO) database (http://www.geneontology.org/, assessed on 28 June 2024) and the KEGG database (http://www.kegg.jp/kegg/, assessed on 28 June 2024). These annotations were used to determine the DEGs’ primary biological functions and identify significantly enriched metabolic and signal transduction pathways.

### 4.6. WGCNA Used to Identify Relevant Gene Networks

A weighted gene co-expression network analysis (WGCNA) was conducted using publicly available R packages, following the standard protocol, with default parameters being used to identify co-expressed genes and proteins. Network construction and module detection were performed using an unsigned topological overlap matrix with a soft-thresholding power of 30 (for unigenes), a minimum module size of 20, and a branch merge cut height of 0.25. Transcripts exhibiting similar expression patterns were clustered into distinct modules, and their eigengenes were subsequently calculated. Finally, phenotypic data were integrated into the WGCNA framework to assess correlations between phenotypic traits and unigene modules.

### 4.7. Validation by qRT-PCR

To validate the RNA-seq results, we selected 4 DEGs for a quantitative real-time PCR (qRT-PCR) analysis using the SYBR green dye method. Primers for qRT-PCR were designed with Primer Premier 6 software and synthesized by Shanghai Yipixin Biotechnology Co., Ltd. (Shanghai, China) (Table S11). Following the manufacturer’s protocol, total RNA was reverse-transcribed into cDNA using the HiScript First Strand cDNA Synthesis Kit (Novizan Biotechnology Co., Ltd., Nanjing, China). The qRT-PCR reactions were conducted using the AceQ qPCR SYBR Green Master Mix kit on a 96-well plate (PCR-96-FLT-C), following the manufacturer’s guidelines. The cycling conditions were as follows: initial denaturation at 95 °C for 5 min, followed by 40 cycles of 10 s at 95 °C and 30 s at 60 °C. The relative changes in unigene expression levels were calculated using the 2^−ΔΔCt^ method. Data are represented as the mean ± standard deviation (S.D.) of three biological replicates.

### 4.8. Statistical Analysis

Fruit phenotypic characteristics, economic traits, and physiological-biochemical parameters were statistically analyzed using a one-way analysis of variance (ANOVA), followed by Duncan’s multiple comparison test (*p* < 0.05) in IBM SPSS Statistics 25.

## 5. Conclusions

In summary, this study comprehensively analyzes sugar metabolism in *C. drupifera* fruit across various developmental stages. We identified and analyzed these unigenes by integrating quantitative measurements and correlation analyses of fruit traits, sugar content, and oil content, alongside transcriptome analysis for key regulatory unigenes involved in sugar metabolism. A total of 402, 412, and 316 DEGs related to carbohydrate metabolism were identified in the NS, FA, and MS stages, respectively. Notably, the carbohydrate metabolism-related DEGs exhibited a pattern of 278 upregulated unigenes and 440 downregulated unigenes. A pathway analysis revealed the activation of pathways associated with energy production and carbon skeleton biosynthesis, concurrent with suppressing pathways involved in antioxidation, carbohydrate synthesis, and specific carbon metabolic processes. Furthermore, WGCNA analysis facilitated the identification and preliminary characterization of key regulatory unigenes involved in sugar metabolism, including *SS*, *SUS*, *HXK2*, *SPS4*, and *ALDH2B4*. These findings offer valuable insights into the dynamic changes and regulatory mechanisms governing sugar metabolism during fruit development in *C. drupifera* and establish a robust theoretical foundation for the rational and efficient utilization of this valuable resource.

## Data Availability

The datasets presented in this study can be found in online repositories. The names of the repository/repositories and accession number(s) can be found: https://www.ncbi.nlm.nih.gov/bioproject/PRJNA1218904, accessed on 4 February 2025.

## Supplementary Materials

The following supporting information can be downloaded at: https://www.mdpi.com/article/10.3390/plants14050817/s1. Figure S1. KEGG enrichment analysis of up-and down-regulated DEGs; Table S1. Changes in phenotypic characteristics at different developmental stages. Table S2. Changes in economic traits at different developmental stages. Table S3. Physiochemical Analysis Changes. Table S4. Correlation analysis between economic traits and physicochemical indicators. Table S5. Fold changes of all differentially expressed genes. Table S6. Differential genes associated with carbohydrate metabolism. Table S7. Upregulated unigenes were significantly enriched in biological pathways related to carbohydrate metabolism. Table S8. Downregulated unigenes were significantly enriched in biological pathways related to carbohydrate metabolism. Table S9. Statistical enrichment analysis of DEGs within the MEgreen module. Table S10. Differential genes associated with carbohydrate metabolism. Table S11. Gene primer sequence.
