# Supplementary figures and images for: Gene Expression Dynamics of Sugar Metabolism and Accumulation During Fruit Ripening in Camellia drupifera
Source: Plants (Basel). 2025 Mar 5;14(5):817. doi: 10.3390/plants14050817 (PMC11901808; doi:10.3390/plants14050817)

## Supplementary Figures

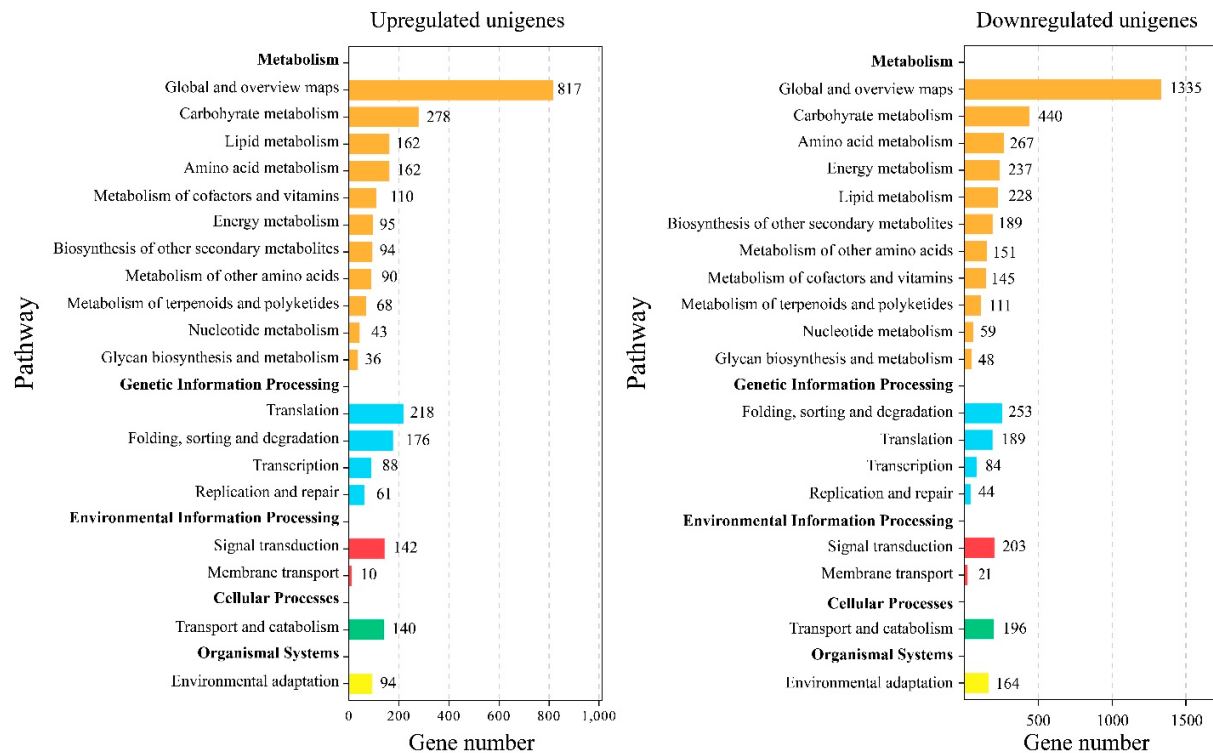

Figure S1. KEGG enrichment analysis of up-and down-regulated DEGs.

Supplement: Supplementary file 1 [file plants-14-00817-s001.zip › Supplementary Figures.pdf]
